# Endoscopic full-thickness retrieval of a migrated plastic stent after endoscopic ultrasonography-guided liver abscess drainage

**DOI:** 10.1055/a-2902-3545

**Published:** 2026-07-16

**Authors:** Kazuya Sumi, Jun Ushio, Hisaki Kato, Yuki Kawasaki, Takayoshi Ito, Yoshio Deguchi, Haruhiro Inoue

**Affiliations:** 1 Digestive Diseases Center 378609 Showa Medical University Koto Toyosu HospitalKoto-kuTokyoJapan

Endoscopic ultrasonography (EUS)-guided interventions are increasingly performed, and the management of procedure-related adverse events is essential. Among these, stent migration into the abdominal cavity often requires surgical intervention, although endoscopic retrieval has occasionally been reported. 1​ 2​ ​3​ We report a case in which a migrated plastic stent (PS) was successfully retrieved using an endoscopic full-thickness approach.

A man in his 80s underwent EUS-guided drainage for a large liver abscess (LA) in the left hepatic lobe. During the procedure, a PS migrated into the abdominal cavity. The LA was subsequently managed by repuncture and the placement of a 6-Fr endoscopic nasobiliary drainage tube, resulting in clinical improvement.

Post-procedural computed tomography demonstrated that the proximal end of the migrated PS was closely apposed to the esophagogastric junction (EGJ) wall in the subdiaphragmatic space. The migrated PS extended from the lesser curvature toward the anterior wall, without intervening major vessels or organs. Following surgical consultation, endoscopic retrieval was planned.

**Fig. 1:**
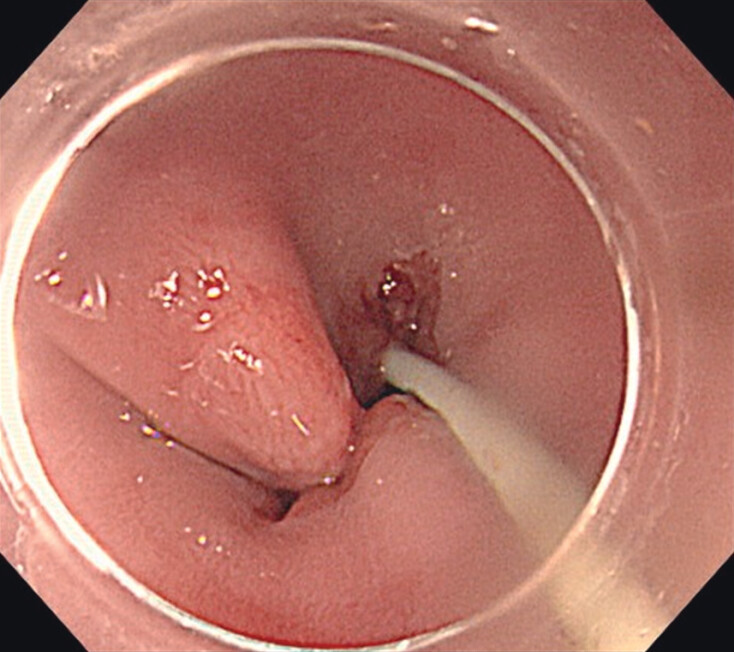
A submucosal tumor (SMT)-like bulge was identified just above the esophagogastric junction (EGJ), near the previously placed external drainage tube.

**Fig. 2:**
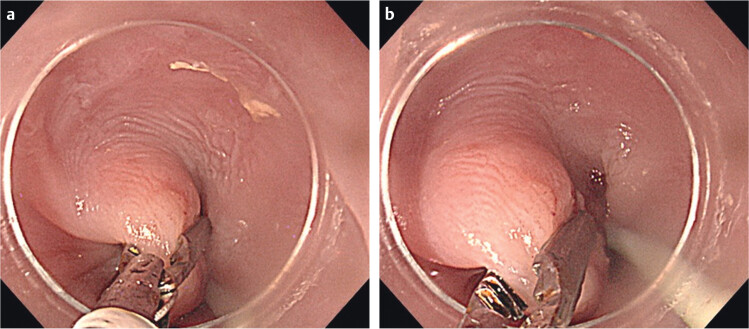
( **a** and **b** ) The bulge was firm when grasped with forceps, and fluoroscopy demonstrated the synchronous movement of the migrated plastic stent (PS).

**Fig. 3:**
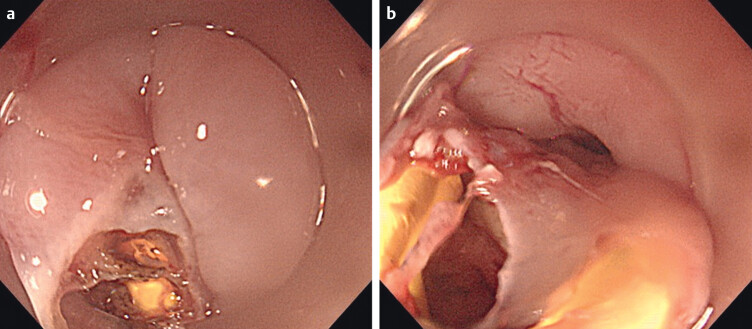
( **a** ) Incision of the mucosa over the bulge provided full-thickness access and exposed the migrated PS. ( **b** ) The stent was grasped with forceps and successfully retrieved under direct visualization.

**Fig. 4:**
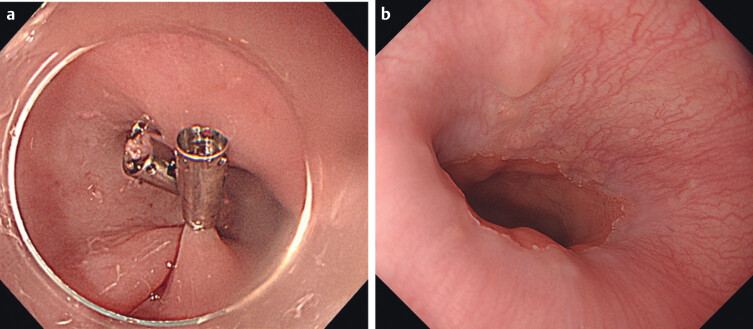
( **a** ) The incision site was closed with clips. ( **b** ) Follow-up endoscopy at 6 months confirmed complete scar formation.

A submucosal tumor (SMT)-like bulge was identified just above the EGJ ( Fig. 1 ), and fluoroscopy confirmed the location of the migrated PS ( Fig. 2 ). The mucosa over the bulge was incised to obtain full-thickness access and expose the PS ( Fig. 3a ), which was then grasped and successfully removed ( Fig. 3b ). The incision site was closed with clips, and the patient was discharged without adverse events. Follow-up endoscopy at 6 months showed complete scar formation ( Fig. 4 and Video 1 ).

**Video 1** Endoscopic full-thickness retrieval of a migrated plastic stent after EUS-guided drainage, allowing safe removal under direct visualization.


In this case, the migrated PS was closely apposed to the gastrointestinal wall and was identifiable as a SMT-like bulge. The approach was feasible because the stent was located along the lesser curvature toward the anterior wall of the stomach, without intervening major vessels or organs. Even in cases of intraperitoneal stent migration, endoscopic full-thickness access may represent a feasible minimally invasive retrieval option when appropriate anatomical conditions are met.

Endoscopy_UCTN_Code_TTT_1AS_2AK
